# Reduction of *Helicobacter pylori* cells in rural water supply using slow sand filtration

**DOI:** 10.1007/s10661-024-12764-2

**Published:** 2024-06-15

**Authors:** Javier Leyton, Javier Fernández, Patricia Acosta, Andrés Quiroga, Francesc Codony

**Affiliations:** 1 Department of Environmental and Sanitary Engineering, Faculty of Civil Engineering, Universidad del Cauca, Popayán, Colombia; 2 Department of Physiological Sciences, Faculty of Health Sciences, Universidad del Cauca, Popayán, Colombia; 3 Municipal Laboratory Aigües de Mataró, Barcelona, Spain

**Keywords:** *Helicobacter pylori*, Emerging pathogens, Drinking water, Slow sand filters, Water quality

## Abstract

*Helicobacter pylori* is a microorganism that infects 60% of the population and is considered the main cause of atrophic gastritis, gastric and duodenal ulcers, and gastric cancer. Different emerging pathogens have been found in drinking water and their presence is considered to be an important public health problem. For this reason, it is necessary to carry out the validation of reliable technologies for this type of pathogens and evaluate their performance. This paper reports, for the first time, *H. pylori* reduction in a drinking water pilot plant of two slow sand filters (SSF). Inlet water was taken from a gravel filtration system of a rural water supply in Colombia and then inoculated with viable cells of *H. pylori*. By determining the Genomic Units (GU) through quantitative Polymerase Chain Reaction (qPCR), the concentration of GU/sample was measured. In the inlet water amplification for SSF1 and SSF2 were 5.13 × 10^2^ ± 4.48 × 10^2^ and 6.59 × 10^2^ ± 7.32 × 10^2^, respectively, while for the treated water they were 7.0 ± 5.6 and 2.05 × 10^1^ ± 2.9 × 10^1^ GU/sample for SSF1 and SSF2, respectively. The SSF pilot plant reached up to 3 log reduction units of *H. pylori*; therefore, since there is not an *H. pylori* contamination indicator and its periodic monitoring is financially complicated, the SSF could guarantee the drinking water quality necessity that exists in rural areas and small municipalities in developing countries, where infection rates and prevalence of this pathogen are high.

## Introduction

*Helicobacter pylori* is a gram-negative bacterium which is considered an emerging pathogen by the EPA’s Contaminant Candidate List (CCL), which lists unregulated contaminants in drinking water of greatest public health concern and research interest (Environmental Protection Agency, n.d., 2021). *H. pylori* is considered the main cause of atrophic gastritis, gastric and duodenal ulcer, and intestinal gastric cancer (Jiménez, 2018; Serrano et al., 2009).

A systematic review carried out by Hooi et al. (2017) revealed that 60% of the global population is infected with *H. pylori* and presents variations in its distribution, while regions with developing countries have the highest prevalence of the infection: Africa (79.1%), Latin America, the Caribbean (63.4%), and Asia (54.7%). Studies report a prevalence of *H. pylori* infection of 83.1% for Colombia (Castillo Cañón, 2014; Porras et al., 2013). In Colombia, the presence of *H. pylori* was related to 13.7% of all gastric cancer deaths in the country, being the first cause of this type of death in men and the third for women. The department of Cauca reports one of the highest mortality rates in the country (Adrada et al., 2008; INC, 2017).

Different epidemiological studies suggest that the fecal–oral and oral-oral are the transmission routes of *H. pylori*. on which contaminated water could play an important role in the spread of the pathogen to humans (Acosta et al., 2015; Bartram & Cairncross, 2010; Goh et al., 2011; Percival & Thomas, 2009; Rolle-Kampczyk et al., 2004; Santiago et al., 2015, 2016; Vesga et al., 2018a). This bacterium has been declared a high global priority pathogen by the WHO for research and development due to its resistance to antibiotics, driving the development of control measures and adequate disinfection strategies (Carreño & Rojas, 2018; WHO, 2017, 2022b). The epidemiological link between the infection by *H. pylori* and the consumption of untreated water has been established as a result of inadequate operation of treatment plants (Acosta et al., 2015), and its infiltration into drinking water distribution systems (Baker et al., 2002; Park et al., 2001; Watson et al., 2004). In addition, Duarte et al. (2021) and Santiago et al. (2015) demonstrated that presence predominant coccoid cells of *H. pylori* in a streams, drinking, and irrigation water is an indicative of the viable but nonculturable (VBNC) state; therefore, the survival of this bacteria in water has been proven.

The presence of *H. pylori* is higher in developing countries and in rural areas, mainly due to lack of access to basic services and poor hygiene practices (Carreño & Rojas, 2018). Additionally, in Colombia, the water treatment processes employed face significant operational constraints, particularly in small municipalities and rural areas where infrastructure either does not exist or is only partially implemented. It is estimated that 76% of the infrastructure constructed for water treatment in rural areas is non-operational (Findeter, 2021). The quality of the water supplied to the population presents some type of risk, as shown by the Water Quality Surveillance report of the National Institute of Health (INS, 2020) where it was reported that based on the quality risk index of the water, the consolidated behavior of the country showed that 27% of the samples analyzed presented some level of risk for consumption. However, if only the self-supplying systems are considered (most of them located in rural areas), the number increases to 68%, showing the great health problems in rural areas.

Different emerging pathogens have been found in drinking water and research highlights that their presence is considered an important public health problem, where even the absence of *Escherichia coli* is not a guarantee of the absence of these pathogens or that their concentrations are below those of significant concern. This is the reason why it is necessary to validate reliable technologies for this type of pathogens and evaluate their performance (Razzolini et al., 2010; WHO, 2022b).

The presence of pathogens in drinking water from conventional treatment plants by chemical coagulation means that there are threats to public health despite receiving treatment (Tahar et al., 2022; Vesga et al., 2018a, 2018b). Due to the high requirements in operation and maintenance this type of technology, there exist significant limitations for its viability in rural areas of developing countries. Hence, multi-stage filtration (MSF) technology could be an alternative for water treatment, and due to its ease of operation, it could be used in these less populated and developed areas (WHO, 2022a, 2022b). Having Slow Sand Filters (SSF) that remove microorganisms such as *Giardia* and *Crystosporidum*, the latter resistant to chlorine disinfection (Betancourt & Rose, 2004; Richard et al., 2016), could be the solution for the reduction of *H. pylori* in rural areas and in small municipalities, where infection rates and prevalence of this bacteria are high.

Ledezma et al. (2024) studied the removal of *H. pylori* in gravel pretreatment systems, used in the rural water supply system of the Los Llanos village in Popayán, Colombia. The raw water source indicated the presence of *H. pylori* in 10% of the samples analyzed. The results showed that there was an increase in the number of positive samples for *H. pylori* (20%) at the treatment system outlet, as well as in the fecal contamination indicators. This poor behavior in microbiological removal, apparently, is associated with inadequate operation and maintenance of the gravel pretreatment system.

In this study, two SSF were designed and supplied with drinking water from the Los Llanos water supply system, Popayán. The objective was to evaluate for the first time the reduction of *H. pylori* through slow sand filtration using quantitative Polymerase Chain Reaction (qPCR).

## Materials and methods

The research approach is quantitative and exploratory in a pilot plant, located in the facilities of the Universidad del Cauca in Popayán, Colombia. The implemented pilot plant used water from the supply system of the Los Llanos, which has a two-stage filtration gravel pretreatment system.

**Fig. 1:**
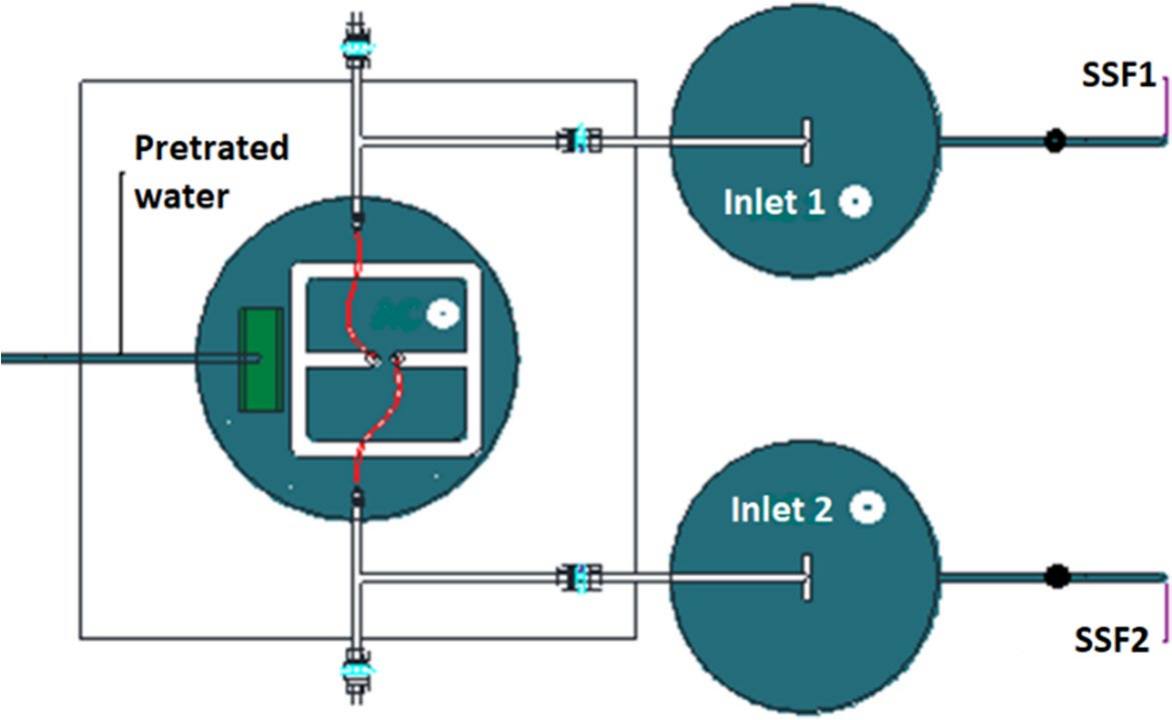
Plant view of pilot plant system

**Fig. 2:**
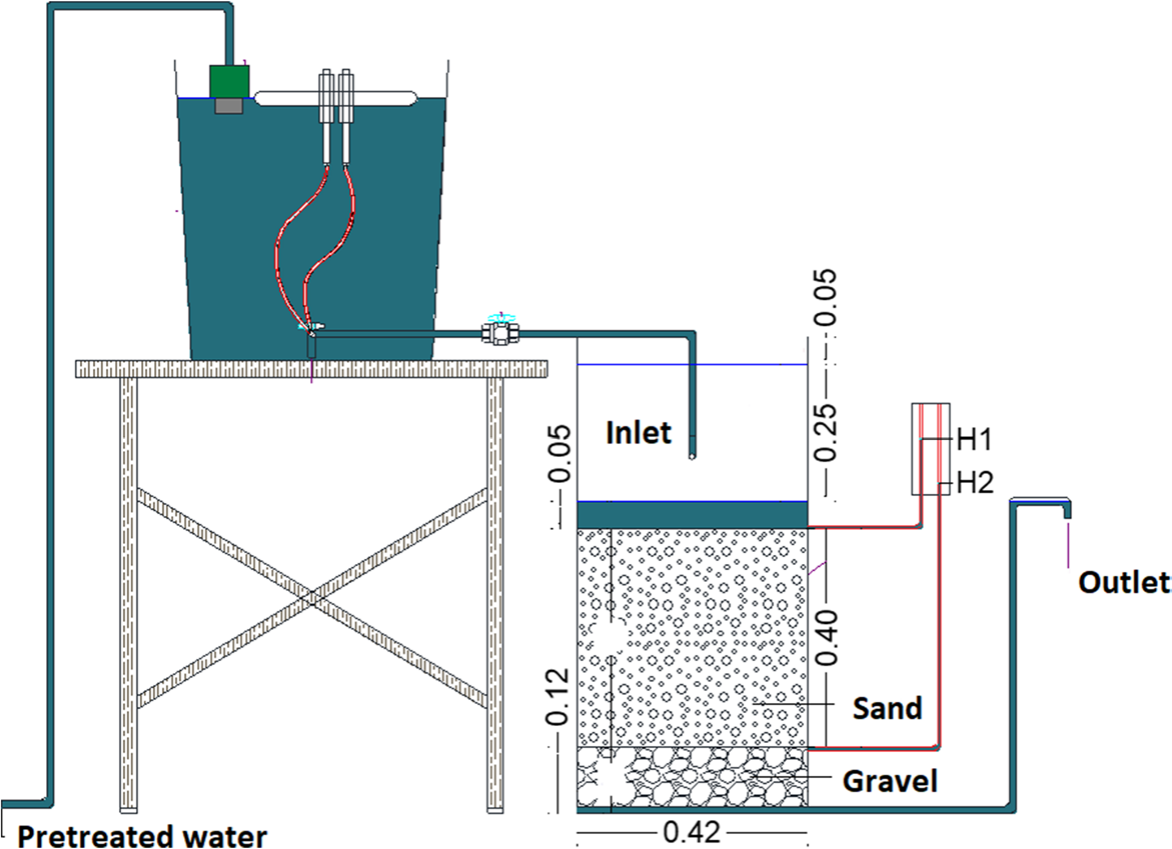
Profile view of pilot plant system

The pilot plant consisted of an 80-l head tank, equipped with a float valve to keep the water level constant; and a constant head dosing system and two identical SSF (see Fig. 1). The filters were made up of cylindrical plastic containers with a diameter of 0.42 m and a height of 0.87 m. The filter bed was conditioned with a 0.40-m layer of sand, with a coefficient of uniformity (CU) of 2.86 and an effective diameter (D10) of 0.22 mm. The profile of the pilot plant is presented in Fig. 2.

The evaluation of the system was carried out using a filtration rate of 0.15 m^3^/m^2^/h, following the recommendations described by Galvis and Latorre (1999) and Sánchez et al. (2007) for SSF design. The system was initially put into operation for a period of 2 months, to facilitate the formation of the “*schmutzdecke*” (biological layer of the sand surface). Subsequently, the surface maintenance of the sand was carried out and it was put into operation to carry out the monitoring. A maximum head loss of 25 cm was considered, which indicated the completion of assessment.

Filter performance was monitored weekly with water quality parameters such as turbidity, color, pH, total coliforms, and *E. coli* measured according to the standard methods for the examination of water and wastewater. Additionally, operational parameters such as flow and head loss were monitored.

*H. pylori* quantification was performed using the quantitative Polymerase Chain Reaction (qPCR) as it will be described above. This molecular technique was used since *H. pylori* can enter its viable but non-culturable cell (VBNC) state under unfavorable conditions. In this state, it can be metabolically active and retain the virulence genes that give it the ability to survive for long periods of time in water (Zambrano Ovalle, 2012; Linke et al., 2010; Park et al., 2001).

### Sample collecting

Although in the studies carried out by Ledezma (2021), *H. pylori* was detected in water pretreated by the gravel filtration system, to ensure its presence in the inlet water of SSF, the inoculation of *H. pylori* was carried out in the supernatant of the filters without interrupting the flow of pretreated water. The preparation of the solution to be inoculated started from the resuspension of cultured viable *H. pylori* cells 11637 and 11638, preserved in BHI broth (Scharlau, Spain), in 1 ml of PBS adjusted to a McFarland scale 1–3. An aliquot of 50–150 µl was diluted in 1 l of water. This volume of solution was introduced and homogenized in each of the filters through a sterile hose. The inlet water samples (pretreated-inoculated water) were taken in sterile containers in the supernatant of each filter, by means of a continuous drip system for a period of 5 min until a volume of 1 l was obtained per each unit.

The treated water samples were taken considering the retention time in the filter, for which a previous tracer study was performed that allowed this time to be determined. The study indicated that the median retention time was 86 min. Therefore, a 1000 ml composite sample was taken for each unit over 30 min, starting 15 min before the average residence time. During this time, 100 ml aliquots were taken every 3 min until the required volume was reached.

This sample collection method was carried out for 8 weeks, during which time the SSF reached the maximum designed head loss level.

### Concentrating *Helicobacter pylori* cells

For each sampling point, 300 ml of homogenized sample were taken in 50 ml centrifuge tubes, which were centrifuged at 4000 rpm, 8 °C for 30 min (Megafuge 1.0R—Heraeus). Subsequently, the supernatant was discarded and approximately 4 ml of concentrated sample from each tube was taken to a second centrifugation in 1.5 ml tubes at 14000 rpm for 5 min (Eppendorf 5415C), the supernatant was discarded and successive centrifugations were made until obtaining two concentrated samples of 200 µl per sampling point. An internal control of the entire process was included for each sampling process, using an additional 50 ml centrifuge tube with pretreated water and 1 µl of the same viable cultured cells used in the inoculation.

### Extraction and purification of DNA

The concentrates were processed using the E.Z.N.A.® Tissue DNA kit (Omega Bio-Tek, Doraville, USA), following the manufacturer’s recommendations and the adaptation made by Acosta et al. (2018). The isolated DNA was eluted in 50 μl of buffer solution and stored at − 30° C until amplification. Molecular grade water was used as an internal extraction control in all tests. All samples were quantified using the Nanodrop 2000 equipment to validate the quality and quantity of the DNA.

### *H. pylori* quantification

The qPCR quantification method was previously developed by Acosta et al. (2018) and verified according to the protocol described in the ISO 12869 standard. Although it focuses on the detection of Legionella spp., the criteria described in Chapter 9 can be applied to any PCR method for water microbiology.

The standard curve was constructed from base 10 serial dilutions of DNA strain NCTC 11638 of known concentration. This was set up in triplicate with a linear range of 6 logarithms.

DNA amplification was carried out using previously reported *H. pylori* specific primers to amplify the allele s of the VacA gene (Atherton et al., 1995; Erzin et al., 2006; Yamaoka et al., 1999). Additionally, the specificity of the qPCR was verified using DNA from three strains of *H. pylori* (TX30a, 11,638, and 11,637). The amplification mix contained 0.5μL of each primer, 10 μl of Sso Advanced Universal SYBR Green with ROX prepared according to the manufacturer’s recommendations, and 5μl of genomic DNA. The strips were briefly centrifuged at low speed to bring the entire sample to the bottom of the tube. PCR-grade water was used as negative control in all qPCR assays. The samples were taken to the thermal cycler in real time QuantStudio™ 3 of Applied Biosystems under the following conditions: one step of 5 min at 96 °C for denaturation, and 45 cycles: 96 °C for 15 s, 57 °C for 20 s, and 70 °C for 50 s. Finally, melting point analysis was performed by raising the temperature slowly (0.1 °C s^−1^) from 60 to 95 °C.

### Statistical analysis

Considering the two filters as repetitions, it was analyzed whether the water quality results had a parametric or non-parametric distribution, and from these results, the statistical differences between the two SSFs were determined. In this case, the data presented a non-parametric distribution, so the Kruskal–Wallis statistical test was used.

## Results

### Water quality

From Table 1, it can be seen that the quality of the inlet water to the pilot plant adjusts to the conditions required by slow sand filters for good performance. The average turbidity and color of the water entering the system are within the recommended limits for waters with turbidities below 10 NTU and colors less than 25 Pt–Co (Galvis & Latorre, 1999; Sánchez et al., 2007). In the same way, some authors suggest an amount less than 1000 CFU/100 ml for total coliforms at the filter’s inlet is also within recommended limits. Although in some cases, maximum values were obtained, it is acceptable, as long as it is for a short period of time that does not exceed two consecutive days (Ferreira et al., 2015; Sánchez et al., 2007).

**Table 1:** Average and ranges of quality water parameters of the inlet and treated by SSF1 and SSF2

Parameters		Inlet	SSF1	SSF2
Turbidity (NTU)	Average	2.26	0.41	0.50
	Range	1.82–2.88	0.26–0.61	0.31–0.84
Color (Pt–Co)	Average	26	13	14
	Range	20–36	6–22	5–21
pH	Range	6.9–7.8	6.9–7.9	7.0–7.9
Total Coliforms (CFU/100 ml)	Average	3088	55	141
	Range	715–12,100	28–110	40–506
*Escherichia coli* (CFU/100 ml)	Average	22	1	0
	Range	10–80	0–8	0–1

According to the Kruskal Wallis statistical test, there is no significant difference between SSF1 and SSF2 for all parameters, since the *P* value is greater than 0.05 (turbidity P: 0.27; color P: 0.79; total coliform P: 0.08; E. coli P: 0.81), with a confidence level of 95%. Thus, effectively, the two filters are replicates of each other.

### Quantitative PCR standardization

**Fig. 3:**
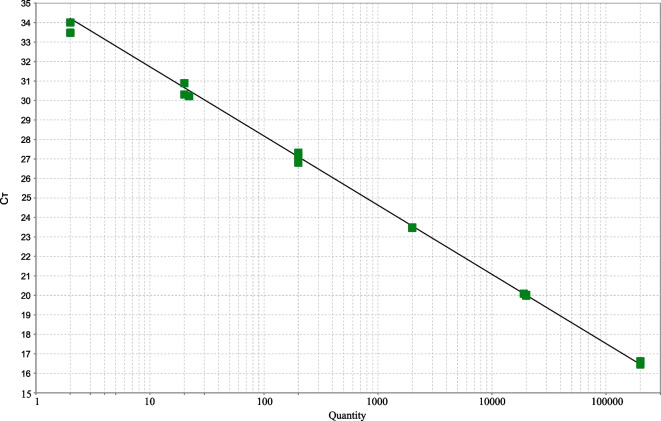
Standard curve used for quantification

The qPCR technique was validated through the parameters of efficiency, specificity, and sensitivity (Bassy Álvarez et al., 2018). The standard curve used had an *R*^2^ of 0.995 and an efficiency of 91%, being within the acceptable range, according to what is mentioned in Applied Biosystems (2006) and Svec et al. (2015). Cycles to Threshold (CT) values between 16.45 and 34 were obtained, corresponding to 2 to 2 × 10^6 GU (see Fig. 3).

**Fig. 4:**
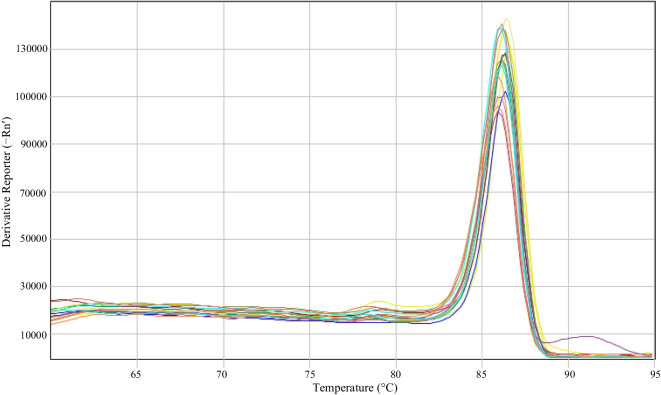
Melting curve plot

The specificity results showed a melting temperature (Tm) of the control strains (85.97–86.62) and of the strains used in the inoculation (86.19–87.49) in accordance with that reported for *H. pylori* (Álvarez & Ceballos, 2018; Castillo Cañón, 2014; Vesga et al., 2018a) (see Fig. 4).

### Quantification of *Helicobacter pylori* cells

Whole inlet water samples for the two filters were positive for *H. pylori*, as expected given the inoculation performed with these cells. Considering the volume used in the concentration of the water samples, the DNA elution volume, and the DNA volume used in the amplification, the concentration factor for a 100 ml volume was determined. It was 10 and 12 GU/100 ml for the treated and inlet water, respectively. From this concentration factor, a detection limit in water of 15 GU/100 ml was obtained. The results are summarized in Table 2.

**Table 2:** *Helicobacter pylori* concentration in the inlet and treated water by SSF1 and SSF2

	Inlet 1	Inlet 2	SSF1	SSF2
GU/100 ml	6151	7910	73	207
*σ*	5377	8783	53	288
Log Reduction	-	-	1.8	1.6
*n*	12	12	12	12

**Fig. 5:**
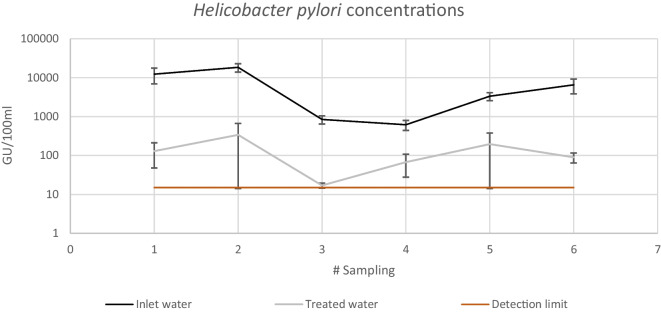
Performance of Helicobacter pylori concentrations at the inlet and outlet of the SSF

Using the Kruskal–Wallis test, the hypothesis that the *H. pylori* reduction medians are the same for the two filters was evaluated. The results show that there is no statistically significant difference between the medians, with a 95% confidence level (*P* = 0.08). Therefore, as expected, the two SSFs were replicates of each other. In Fig. 5, the monitoring of *H. pylori* concentrations in the inlet and outlet water of the SSFs system are shown.

Figure 5 shows a reduction of *H. pylori* cells for the two systems, with a logarithmic reduction of 1.7 ± 0.5 and the detection limit of 15 UG/100 ml. In 50% of the sampling, at least one sample taken into the effluent of the SSFs were below the detection limit, as seen in the scatter bars of the data; therefore, the log reduction could be greater than the average shown.

## Discussion

In general, the efficiency of the treatment by slow sand filtration performs as expected for this type of system (Ferreira et al., 2015; Sánchez et al., 2007). The percentage of removal of turbidity and apparent color was 79.3 ± 7.8 and 48.8 ± 15, respectively. For total coliforms, the logarithmic reduction was 1.4 ± 0.4, a similar reduction to that reported in the reference literature (Aguirre Osorio, 2012; Collins, 2000; Abdiyev et al., 2023; Ramírez Medina, 2011); and for *E. coli*, due to the number of samples of treated water with zero colonies, removal is considered almost 100%, which is expected for this type of system (Bellamy et al., 1985; Cánepa De Vargas, n.d.; Villanueva Perdomo, 2013). The behavior described above shows that the SSFs are performing adequately in the removal of physical parameters and microbiological indicators, so the biological layer (schmutzdecke) is well constituted and mature, making it possible to validate the reduction of the emerging pathogen *H. pylori* under normal operating conditions.

The number of *H. pylori* genomic units per sample (GU/sample) detected in the inlet water for SSF1 and SSF2 were 5.13 × 10^2^ ± 4.48 × 10^2^ and 6.59 × 10^2^ ± 7.32 × 10^2^, respectively. Similar results were reported by Vesga et al. (2018a) in raw water samples, with values between 1.28 × 10^1^ and 4.69 × 10^2^ GU/sample. Likewise, Santiago et al. (2015) quantified the presence of *H. pylori* and obtained concentration values of 5.54 × 10^2^ and 1.59 × 10^3^ GU/sample in drinking water supplies. However, in both author’s works, these results do not represent the real level of water contamination, since their methodology included a pre-enrichment step.

The experimental detection limit of 15 UG/100 ml, showed to be below previous reports found for drinking water (Boehnke et al., 2018), so the previous standardization of the technique facilitated to achieve greater sensitivity to enable the detection of *H. pylori* at the effluent of the SSF and to determine removal efficiencies.

For the treated water, the *H. pylori* concentrations were 7.0 ± 5.6 and 2.05 × 10^1^ ± 2.9 × 10^1^ GU/sample for SSF1 and SSF2, respectively. Vesga et al. (2018a) also reports the *H. pylori* quantity at the outlet of the water treatment plants and after chlorination with concentration values that ranged between 5.77 and 2.12 × 10^3^ GU/sample. Similarly, studies carried out in Panama, Costa Rica, Perú show concentrations in drinking water up to 3.6 × 10^3^ UG/100 ml, even after disinfection (Boehnke et al., 2018; Montero Campos et al., 2015). These reported values show little to no pathogen reduction for the treatment evaluated and after disinfection. Therefore, in this study *H. pylori* concentration at the outlet of the SSF treatment show lower values than those reported for drinking water, highlighting that, in our case, the treated water has not undergone a disinfection process. On the other hand, in the SSFs evaluation (4) four filtered samples did not amplify, so their log reduction was up to 3 logs.

Although with the present study the possibility of reduction of *H. pylori* by SSF is verified, its removal mechanism is not clear. However, given the conditions of the SSF, the mechanism could be of biological due to predation and sweeping by protozoa, rotifers, and macroinvertebrates that feed on organic matter present in the filter bed, considering the ecosystem that is generated in the biological layer of the filter and its great activity, as has been previously described (Haig, 2014). In the same way, the bacteria could be removed by the adsorption mechanism, where it is agglutinated when in contact with the “schmutzdecke” that covers the surface of the sand. Considering the predilection of *H. pylori* to grow in biofilms under unfavorable environmental conditions (Zambrano Ovalle, 2012; Bomo et al., 2003; Linke et al., 2010; Park et al., 2001). In addition, considering that the spiral form of *H. pylori* is 3 µm long and 0.5 µm in diameter, even smaller in its coccoid or VBNC form (Krzyżek et al., 2019), its removal becomes inefficient through other mechanisms present in filtration, such as sieving and sedimentation.

Reductions by slow sand filters have been reported for other pathogens such as helminth eggs, *Fasciola hepatica* eggs, enterobacteria, enteroviruses, Schistosome cercariae, protozoan cysts such as of *Cryptosporidium* and *Giardia* (Aguirre Osorio, 2012; Cánepa De Vargas, n.d.; Collins, 2000; Galvis & Latorre, 1999; Haig, 2014; Maciej Serda et al., 2013; Abdiyev et al., 2023), being the last two most frequent causes of diarrheal infections and associated with numerous global waterborne outbreaks (Efstratiou et al., 2017; Lim & Nissapatorn, 2017). Therefore, this is the first study that demonstrates the reduction of the emerging pathogen *H. pylori* cells in slow sand filters. These results have potential applications on the supply of quality water needs in small municipalities and rural areas of developing countries where the main shortcomings are located.

Normally, the monitoring and surveillance of the microbiological quality of water is assessed with indicators of fecal contamination such as *E. coli*, however, the WHO mentions that “*Escherichia coli* (or, alternatively, thermotolerant coliforms) is not a reliable indicator for the presence/absence of *Helicobacter pylori*” (WHO, 2022b). Similarly, studies carried out by Ledezma (2021) and Vesga et al. (2019) provide evidence of the presence of *H. pylori* in raw and drinking water and show that the detection and quantification of fecal indicator bacteria and physicochemical parameters in water do not correlate with the risk of contamination with *H. pylori*. Therefore, due to not having a good indicator of the presence of *H. pylori* in drinking water and the economic difficulty associated to its continuous monitoring, the SSF can be a reliable drinking water treatment system for the reduction of emerging pathogens, particularly in disadvantaged areas.

## Conclusion

The SSF pilot plant is capable of removing up to 3 logarithms (99.9%) of *H. pylori*, so it is possible that SSF technology can guarantee drinking water with little to no presence of this emerging pathogen.

Since water does not have an indicator of contamination by *H. pylori* and its periodic monitoring is financially complicated, the SSF could satisfy the quality needs of drinking water that exist in rural areas and small municipalities in developing countries, where infection rates and prevalence of this bacteria are high.

It is recommended to continue with the sampling and studies that extend the results contained in this work on the reduction of *H. pylori* at filtration rates greater than 0.15 m^3^/m^2^/h, and to study the reduction of *H. pylori* in SSF that use non-woven geotextile on the surface of the filter bed to improve removal efficiency and generate longer filtration runs, as well as use higher and more variable filtration velocity.

## Data Availability

Data published in this study are available on request to the corresponding author.
